# Valorisation of Mangrove Leaf Powders: Physicochemical Characterisation and FT-MIR Monitoring of Linseed Oil Stability

**DOI:** 10.3390/foods15203724

**Published:** 2026-10-19

**Authors:** Manjeet Singh, Eliot Botosoa, Romdhane Karoui

**Affiliations:** University of Artois, University of Lille, University of Littoral Côte d’Opale, University of Picardie Jules Verne, University of de Liège, INRAE, Junia, UMR-T 1158, BioEcoAgro, F-62300 Lens, France; majeet_singh@ens.univ-artois.fr (M.S.); herinirina.botosoa@univ-artois.fr (E.B.)

**Keywords:** FT-MIR, linseed oil, oxidation, polyphenols, mangrove, chemometrics

## Abstract

This study investigated Fourier-transform mid-infrared (FT-MIR) spectroscopy coupled with chemometric tools as a rapid, non-destructive approach for monitoring oxidation in linseed oil supplemented with mangrove-derived extracts. Young and mature leaves of *Avicennia marina* and *Rhizophora mucronata* were characterised for water activity, moisture, pH, protein, ash, mineral composition, and colour, providing a comparative raw-material profile before extract preparation. The corresponding extracts were evaluated in linseed oil during a 15-day Schaal oven test, alongside butylated hydroxytoluene (BHT), rosemary extract, and an untreated control. FT-MIR spectroscopy was used to follow changes in three spectral regions: 1200–1150 cm^−1^, associated with the C–O/ester-related band; 1742–1720 cm^−1^, linked with the carbonyl interval; and 1532–1510 cm^−1^, related to aromatic compounds. Chemometric modelling was intentionally restricted to the 1200–1150 and 1742–1720 cm^−1^ intervals to assess storage-related variation in the C–O/ester and carbonyl features of the oil matrix. Factorial discriminant analysis (FDA) applied to the first five principal components of the principal component analysis (PCA) of each spectra region achieved leave-one-spectrum-out classification accuracies of 58.10% and 71.43% for the C–O/ester-related and carbonyl models, respectively. The FDA applied to the concatenated both oxidation-related regions improving classification to 76.19%. The combined model captured storage-related spectral evolution more effectively than either individual region or revealed treatment-dependent trajectories relative to untreated oil. These findings complemented conventional oxidation indices and show that targeted FT-MIR spectroscopy coupled with chemometric tools provide a practical framework for monitoring oxidative changes and screening plant-derived preservation strategies in edible-oil systems.

## 1. Introduction

Demand for natural antioxidants is increasing owing to growing consumer awareness of health concerns associated with synthetic antioxidants and the value of plant-derived antioxidants [1,2]. Mangrove plants, commonly found in tropical and subtropical coastal forests, have become an important source of bioactive compounds because of their richness in phytochemicals. Mangrove plants, specifically their leaves, have been shown in numerous studies to contain high concentrations of bioactive phytochemicals such as phenolics, flavonoids, and triterpenoids, possessing powerful antioxidant, antifungal, and anti-inflammatory effects [3,4]. These properties make the resulting biomolecules derived from mangroves valuable and worthy of consideration for use in functional foods, particularly in combating lipid oxidation, specifically because they have potent antioxidant properties [5]. In the search for useful biomolecules against lipid oxidation, the mangrove species *Rhizophora mucronata* and *Avicennia marina* were selected. These species are the most dominant but least studied in Madagascar. Beyond geographic variability, *R. mucronata* is known to exhibit higher phenolic content in nutrient-rich environments such as Indonesia, while *A. marina* shows marked phytochemical variability in response to salinity and ecological conditions across Australian and Indian ecosystems [3]. Although chemical indices such as peroxide value, thiobarbituric acid reactive substances, acid index, and p-anisidine value are commonly employed to assess lipid oxidation and, indirectly, the effects of antioxidants in foods, these methods are time-consuming, thereby limiting their application for routine quality evaluation and process optimisation in food systems. In this context, Fourier transform mid-infrared (FT-MIR) spectroscopy, particularly when combined with chemometrics, has emerged as a highly efficient approach for near real-time monitoring of oxidative changes in food matrices, meeting the need for high-throughput, minimally invasive methods to evaluate food quality, optimise processing parameters, and support sustainable food innovation [6]. FT-MIR spectroscopy measures the vibrational behaviour of chemical bonds within the macromolecules (lipid, protein, carbohydrates, etc.). For example, during oil oxidation, the formation and transformation of oxygen-containing functional groups, together with changes in unsaturation and molecular interactions, modify the infrared absorption profile of the oil. These spectral responses provide a molecular fingerprint of oxidation-related changes and enable their rapid monitoring through chemometric analysis [6,7]. However, the technique reflects overall changes in the molecular environment and does not, by itself, confirm the identity or concentration of individual oxidation compounds.

The polyphenolic compounds in mangroves, such as gallic, caffeic, and ellagic acids, contain multiple hydroxyl groups, enabling them to effectively donate hydrogen atoms and counter lipid-derived free radicals [5,8]. Flavonoids, especially quercetin and catechins, act in synergy with tannins and other phenolic acids in retarding the formation of hydroperoxides and secondary oxidation products through their cooperative action. Metal-ion chelation, especially of Fe^2+^ and Cu^2+^, exerts an additive effect in the suppression of Fenton reaction-mediated reactive oxygen species, despite the occasional pro-oxidative role played by chelated metals in the extracts [5]. Lipid oxidation, particularly in the primary and secondary stages, has long been regarded as a prominent cause of quality deterioration and the eventual development of potentially toxic oxidation products in food [8]. Naturally occurring antioxidants, particularly polyphenols from mangroves, have useful radical-preventing, metal-chelating and enzyme-inhibiting activities that depend on their chemical structure, concentration, and the type of food matrix used [9,10]. The high linoleic acid composition in linseed oil, specifically the alpha linolenic component, tends to make it prone to oxidation. Chemical preservatives such as butylated hydroxytoluene (BHT) and butylated hydroxyanisole (BHA) are widely used for oil preservation, but consumer demand for organic food and natural preservation methods has drawn attention to mangrove phytochemicals [11,12]. Therefore, in this context, the role of polyphenol-rich mangroves like *R. mucronata* and *A. marina*, commonly available in tropical and subtropical conditions, becomes particularly prominent in the present scenario.

In our previous study, the antioxidant efficacy of *A. marina* and *R. mucronata* leaf extracts in linseed oil was evaluated using total phenolic content, antioxidant-capacity assays, conventional oxidation indices, and fluorescence spectroscopy [4]. However, the use of targeted FT-MIR spectra regions coupled with chemometric modelling to follow oxidation-related spectral evolution in mangrove-extract-treated oil has not been examined. The present study therefore had two objectives: first, to document physicochemical, colour, and mineral variation among the leaf powders used for extraction; and second, to assess whether targeted FT-MIR spectroscopy coupled with chemometric tools such as principal components analysis (PCA) and factorial discriminant analysis (FDA) could describe storage-related spectral changes in the same linseed-oil batches. The raw-material measurements provide a traceable comparison of the botanical materials and were not intended to establish direct causal associations with oil stability.

Linseed oil is especially prone to oxidation because of its high alpha-linolenic acid content [8]. Recent accelerated-oxidation studies likewise show that the stability of cold-pressed flaxseed oil depends on its antioxidant composition, additive concentration, and storage temperature [13,14]. This study uses mangrove-extract-treated linseed oil as a model to examine oxidation-related spectral changes by FT-MIR.

## 2. Materials and Methods

### 2.1. Sample Collection and Location

**Figure 1:**
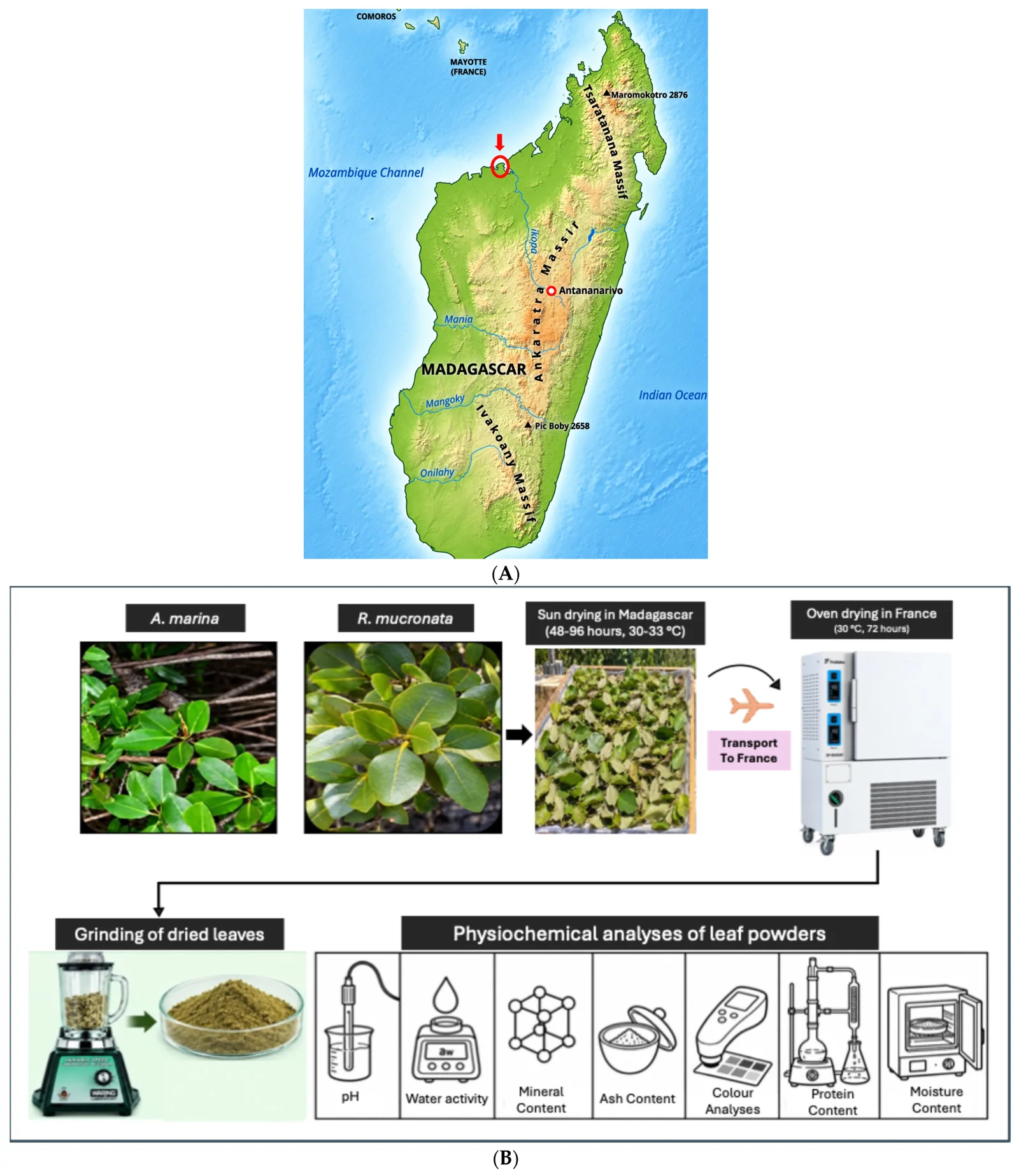

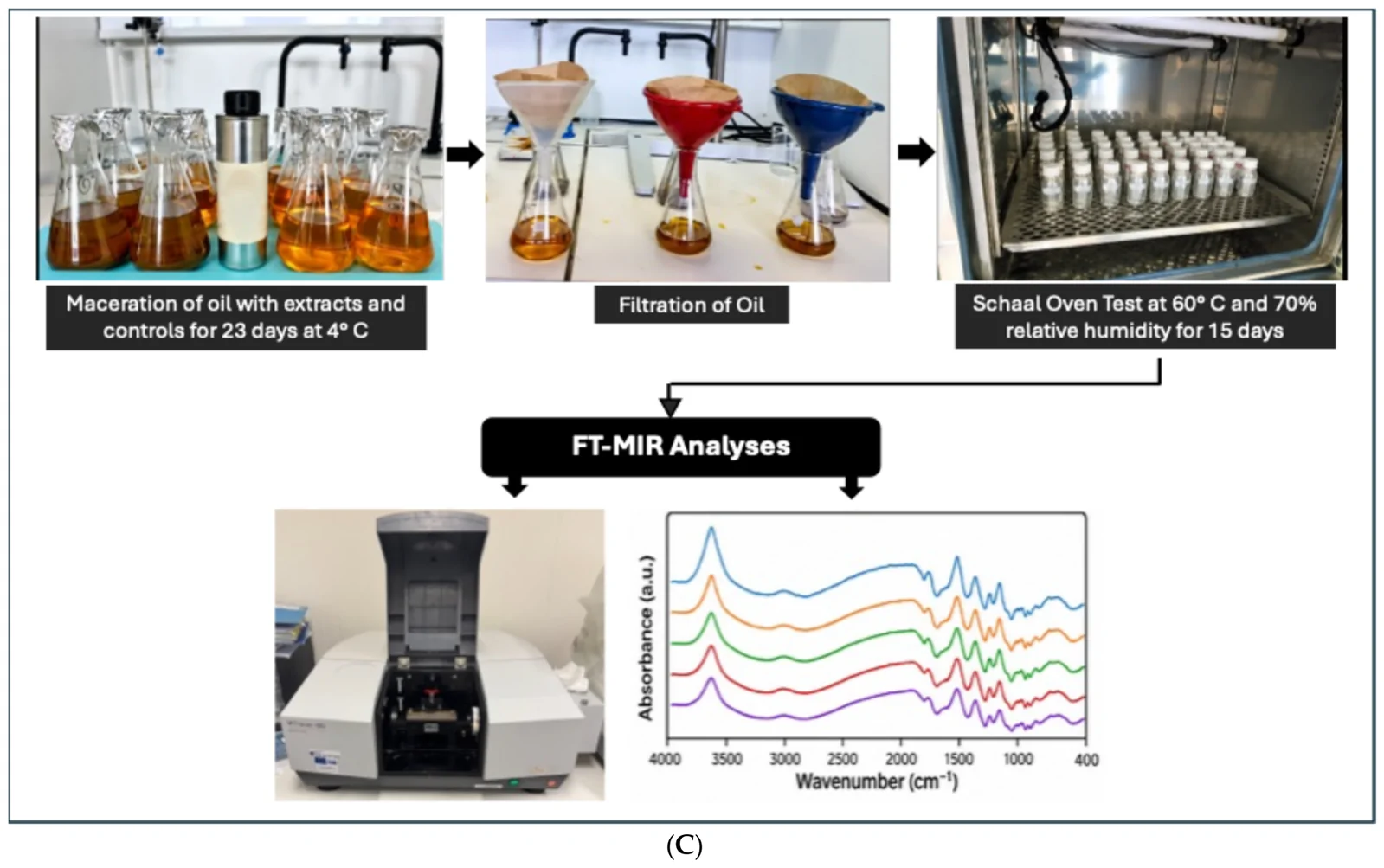
(**A**) Location of mangrove leaf collection sites in Antrema, Madagascar. Source: Singh et al. [4]. (**B**) Sample preparation for physicochemical analyses. (**C**) Schaal oven test, physicochemical analyses, and FT-MIR analyses.

In this study, the mangrove leaf samples previously described by Singh et al. [4] were used for further physicochemical characterisation and FT-MIR analysis. The samples comprised young and mature leaves of *Avicennia marina* and *Rhizophora mucronata*, collected from Antrema, Madagascar (15°43′ S, 46°10′ E; Figure 1A), between May and June 2023.

Immediately post-harvest, the leaves underwent preliminary dehydration via solar drying under ambient conditions (30–33 °C) to reduce their moisture content. Due to intrinsic morphological differences, *A. marina* leaves, characterised by a thinner lamina and lower water content, reached complete desiccation within 48 h, whereas *R. mucronata* leaves, possessing a higher hydration capacity, required 96 h for optimal drying. To promote homogeneous water loss, the leaves were arranged in a single layer and manually turned every four h. After the initial dehydration phase, the samples were subjected to controlled oven drying (Froilabo, model SP-BVEHF, Collégien, France) at 35 °C for 9 days, ensuring further moisture reduction and sample stabilisation prior to transport to France. Upon arrival, an additional post-dehydration step was performed at 30 °C for 72 h to eliminate any residual hygroscopic moisture absorbed during transit. The processed samples were subsequently stored under airtight, dark conditions to prevent oxidative degradation and maintain phytochemical integrity until further analyses.

### 2.2. Physicochemical Analysis

The dried leaves were ground into a fine powder using a grinder (Waring, Torrington, CT, USA) for 90 s. The powder was later used to carry out various analyses, including pH, water activity (aw), colour, and ash, moisture, mineral, and protein contents as indicated in Figure 1B. All analyses were performed in triplicate.

#### 2.2.1. pH Measurement

The procedure began by weighing 5 g of each of the four different mangrove powder samples and transferring them into a flask. Then, 100 mL of distilled water was added, and the mixture was agitated at 500 rpm for 20 min. Subsequently, the samples were allowed to settle for 1 h. Calibration of the pH meter (WTW pH 330i taschen-pH meter, WTW GmbH, Troistedt, Germany) was performed using standard buffer solutions of pH 7.00 and 4.00 at 25 °C.

#### 2.2.2. Water Activity

Water activity (a_w_) was measured at 25 °C using an aw meter (Aqualab, Pullman, WA, USA). The instrument was first calibrated using water activity standards (saturated sodium chloride solution) ranging from 0.25 to 0.50 (a_w_). Each mangrove powder sample was then analysed in triplicate.

#### 2.2.3. Moisture Content

The moisture content of the sample was determined using an oven (Air concept, Firlabo, Collegien, France). For each mangrove leaf sample, 1 g of powder was placed in the oven, with three replicates at 105 °C, until the weight stabilised. The difference in mass before and after drying was then used to determine the moisture content of each sample.

#### 2.2.4. Total Ash Content

The ash content was determined using a muffle furnace. A mass of 0.5 g of each sample was placed in the furnace for 5.5 h at 550 °C [15].

#### 2.2.5. Protein Content

Protein quantification was performed using the Kjeldahl method [16]. The nitrogen content was converted to protein using the conversion factor 6.25, ensuring accurate determination.

(1) $$\mathrm{Protein}\;\mathrm{Content}\;(\%)=\left[\left(V1-V2\right)\times N\times 14.007\times \mathrm{CF}/W\right]\times 100$$

where W = sample weight (g); V1 = volume of 0.1 N HCl used for titration; V2 = volume of HCl used for blank; N = normality of HCl; CF = conversion factor (6.25).

#### 2.2.6. Mineral Composition

For mineral analysis, mangrove powder samples were digested using an Anton Paar system (Les Ulis, France). Each digestion tube contained 0.5 g of powder, 6 mL HNO_3_ (65%) and 1 mL Milli-Q water and was processed for 55 min at 220 °C. After digestion, samples were transferred to Falcon tubes and diluted with Milli-Q water to a final volume of 50 mL. Calibration standards for Ca, K and Mg were prepared at 30, 20, 15, 10, 5 and 2.5 mg/L; for Fe at 2, 1, 0.5 and 0.25 mg/L; and for Cu, Mn and Zn at 3, 2, 1, 0.5, 0.25 and 0.125 mg/L, using 500 and 50 mg/L stock solutions in 5% HNO_3_ (Agilent Technologies, Les Ulis, France). Mineral contents were determined by MP-AES (Agilent 4210) at 213.857, 279.553, 324.754, 371.993, 403.307, 422.673 and 769.897 nm for Zn, Mg, Cu, Fe, Mn, Ca and K, respectively, using an autosampler.

#### 2.2.7. Colour

The colour of the mangrove leaf powder samples was analysed at room temperature (20 °C) using a colourimeter (Minolta Chroma Meter, Model CR-300, Konica Minolta Sensing Europe, Roissy-en-France, France) in the CIE system, where L* indicates lightness, a* is the red/green coordinate and b* is the yellow/blue coordinate. Initially, the instrument was calibrated using black and transparent calibration tools. The transparent sample plates were then filled with the samples, covered and placed on the stage for measurement. This approach allowed for precise determination of the colour characteristics of the mangrove leaf powder samples.

### 2.3. Defatting and Extraction

Dried leaves (packed in tight bags) were defatted with n-hexane (1:5, *w*/*v*) using a mixer (Waring, France) for 3 min, and this procedure was repeated twice. Defatted samples were air-dried under a fume hood for 48 h and stored at 20 °C [17]. For extraction, 4 g of defatted powder was mixed with 40 mL of ethanol, water, and acetic acid (70:30:1) and subjected to ultrasonic extraction (UAE) at 25 kHz, 60% power for 45 min. After filtration, a second extraction was performed with ethanol, water, and acetic acid (90:10:1). Extracts were centrifuged, concentrated at 38 °C [18], and purified by nitrogen evaporation for 5 h, yielding a final product rich in bioactive compounds, i.e., the extract.

### 2.4. Samples and Oil Preparation for Schaal Oven Test

Cold-pressed linseed oil was supplemented with extracts of young and mature *Avicennia marina* and *Rhizophora mucronata* (20 mg dry extract per 200 mL oil), along with butylated hydroxytoluene (BHT) (20 mg in 200 mL oil), rosemary extract (20 mg in 200 mL oil), and a control. Owing to the high viscosity of the botanical extracts, the oil–extract mixtures were stirred and macerated at 4 °C for 23 days to ensure homogeneous solubilisation, followed by filtration and bottling (35 mL oil, 15 mL headspace). Samples were then incubated at 60 °C and 70% relative humidity under Schaal oven conditions (15 days).

The same linseed-oil batches previously characterised by physicochemical and fluorescence analyses in Singh et al. [4] were subsequently analysed by FT-MIR spectroscopy in the present work. The seven treatments were designated as NEG (untreated oil), POS (BHT), ROS (rosemary extract), AJE and AME (young and mature *A. marina* leaf extracts), and RJE and RME (young and mature *R. mucronata* leaf extracts); spectra were acquired on days 0, 3, 7, 11 and 15.

### 2.5. FT-MIR Spectroscopy for Monitoring Oxidation in Linseed Oil

FT-MIR spectroscopy was applied to linseed oil containing mangrove leaf extracts to monitor oxidation-related changes in functional groups during accelerated storage. An IRTracer-100 FT-MIR spectrometer (Shimadzu, Duisburg, Germany) fitted with an attenuated total reflection (ATR) accessory equipped with a grip (Pike Technologies, Inc., Madison, WI, USA) was used to collect infrared spectra. The spectra were recorded in the wavenumber range of 4000–600 cm^−1^ with a resolution of 4 cm^−1^. The ZnSe crystal was carefully cleaned using ethanol and ultra-pure water for each measurement. Spectra were acquired and processed using LabSolutions IR software, IR 2.31 (Shimadzu, Duisburg, Germany). Three technical replicate spectra were acquired for each treatment at each storage day.

### 2.6. Multivariate Statistical Analyses

Physicochemical parameters were compared using analysis of variance (ANOVA). FT-MIR spectra were normalised before applying PCA, separately to the two spectral regions: 1200–1150 cm^−1^ and 1742–1720 cm^−1^. For each spectral region, the first five principal components of the PCA containing more than 99% of the total variance were used for FDA [19,20,21]. The FDA was performed separately for each interval and the concatenated principal-component scores from both intervals to classify spectra according to storage day [22]. The dataset comprised 105 individual spectra obtained from seven treatments, five storage days and three technical replicate acquisitions per treatment–day condition. Leave-one-spectrum-out cross-validation was used as internal exploratory validation; therefore, classification results were interpreted as spectral discrimination rather than external predictive performance. ANOVA and FDA were performed using XLSTAT 2014 software (Addinsoft SARL, New York, NY, USA), while PCA was conducted using MATLAB software (Version 6.5, Release 12, The MathWorks).

## 3. Results and Discussion

### 3.1. Physicochemical Measurements

The physicochemical measurements provide descriptive information on the leaf powders used for extraction and were not used to establish causal relationships with the oxidative stability of the oil. Physicochemical properties of mangrove leaves were highly variable between species and among maturity stages, reflecting their biochemical makeup, metabolic activities, and environmental adaptations. Leaf maturity significantly influenced pH in both *A. marina* and *R. mucronata*, with mature leaves showing significant higher pH values (*p* < 0.05) than young leaves. Mature *A. marina* had the highest pH value (5.93), while young *R. mucronata* leaves exhibited the lowest one (5.007) (Table 1A). This variation in pH would most likely arise from differences in organic acid metabolism, accumulation of phenolic compounds, and ion homeostasis mechanisms, particularly in response to fluctuating salinity and tidal flooding [23]. Younger leaves are metabolically more active and hence likely to show higher rates of organic acid biosynthesis, for example, of malic and citric acids, which tend to decrease pH. The shift to more alkaline pH in mature leaves can be explained by a higher content of inorganic cations, including calcium, magnesium, and potassium, which buffer acidity [24].

Interestingly, the obtained results are in line with those observed for mineral composition analysis. For *A. marina*, there was a significant increase in Mg concentrations from 35.33 mg/L in young leaves to 45.115 mg/L in mature ones, while in *R. mucronata*, there was a lesser but still significant increase from 34.015 mg/L in young leaves to 35.91 mg/L in mature leaves (*p* < 0.05). In both species, Fe levels also significantly increased with leaf maturity from 1.385 to 1.655 mg/L in *A. marina* and from 1.22 to 1.885 mg/L in *R. mucronata* (*p* < 0.05) [25,26]. These data describe maturity-related variation in mineral composition. Notably, young *A. marina* and *R. mucronata* had the highest K levels (101.01 mg/L and 69.04 mg/L), critical for osmoregulation and enzymatic reactions, especially in saline environments where osmotic stress is one of the main drivers of leaf physiology [27].

Water activity (a_w_) showed significant differences between species, indicating differences among species and maturity stages (Table 1A). Mature *R. mucronata* had the lowest a_w_ (0.362), while young *A. marina* and *R. mucronata* leaves presented the highest a_w_ values (0.386 and 0.384, respectively). The lower a_w_ in mature leaves may be due to the greater deposition of lignin and secondary metabolites such as tannins and flavonoids, which reduce the availability of free water and increase the structural rigidity [28]. The moisture content of the dried leaf powders ranged from 1.02 to 1.24 g/100 g. *R. mucronata* young leaves exhibited a significantly higher moisture content (1.24 g/100 g) than *A. marina* young (1.05 g/100 g) and mature leaves (1.02 g/100 g) (*p* < 0.05), whereas no significant difference was observed between *A. marina* young and mature leaves or between *R. mucronata* mature leaves (1.09 g/100 g) and the other samples sharing common superscript letters. Overall, the low residual moisture across all samples confirms effective dehydration prior to analysis (Table 1A).

Young *A. marina* showed the highest protein content (11.07 g/100 g), consistent with the findings of Jamarun et al. [29] (Table 1A). Similarly, young *A. marina* exhibited the highest ash content (13.15 g/100 g), reflecting differences in inorganic residue among the leaf powders [30].

The colour parameters of mangrove leaves provided additional insight into leaf visual traits and potential pigment balance under coastal environmental conditions (Table 1C). Mature *A. marina* leaves showed the highest lightness (L = 55.11) and hue angle (h = 90.92), corresponding to a lighter surface with a yellow-green tonality compared with the other samples, consistent with the CIELAB system, where L* represents lightness and hue angles around 90° indicate yellow-green colours. In contrast, mature *R. mucronata* leaves exhibited the highest b* value (29.41) and chroma (C* = 29.98), indicating a more saturated yellow-green appearance, as positive b* values and higher C* denote stronger yellow components and greater colour intensity. These colour differences describe visual variation among the powders.

Such findings indicate species-specific ecophysiological adaptations of mangroves to saline and tidal habitats, with implications for coastal resilience, nutrient cycling and food valorisation. The findings suggest that young mangrove leaves, particularly those of *A. marina*, may be a good source of protein-rich food additives or functional ingredients, while mature *R. mucronata* leaves, with their high flavonoid and carotenoid content, could serve as potent antioxidant sources for food preservation and nutraceutical applications [31].

### 3.2. Correlation Patterns Linking Physicochemical, Mineral, and Colour Measurements

**Figure 2:**
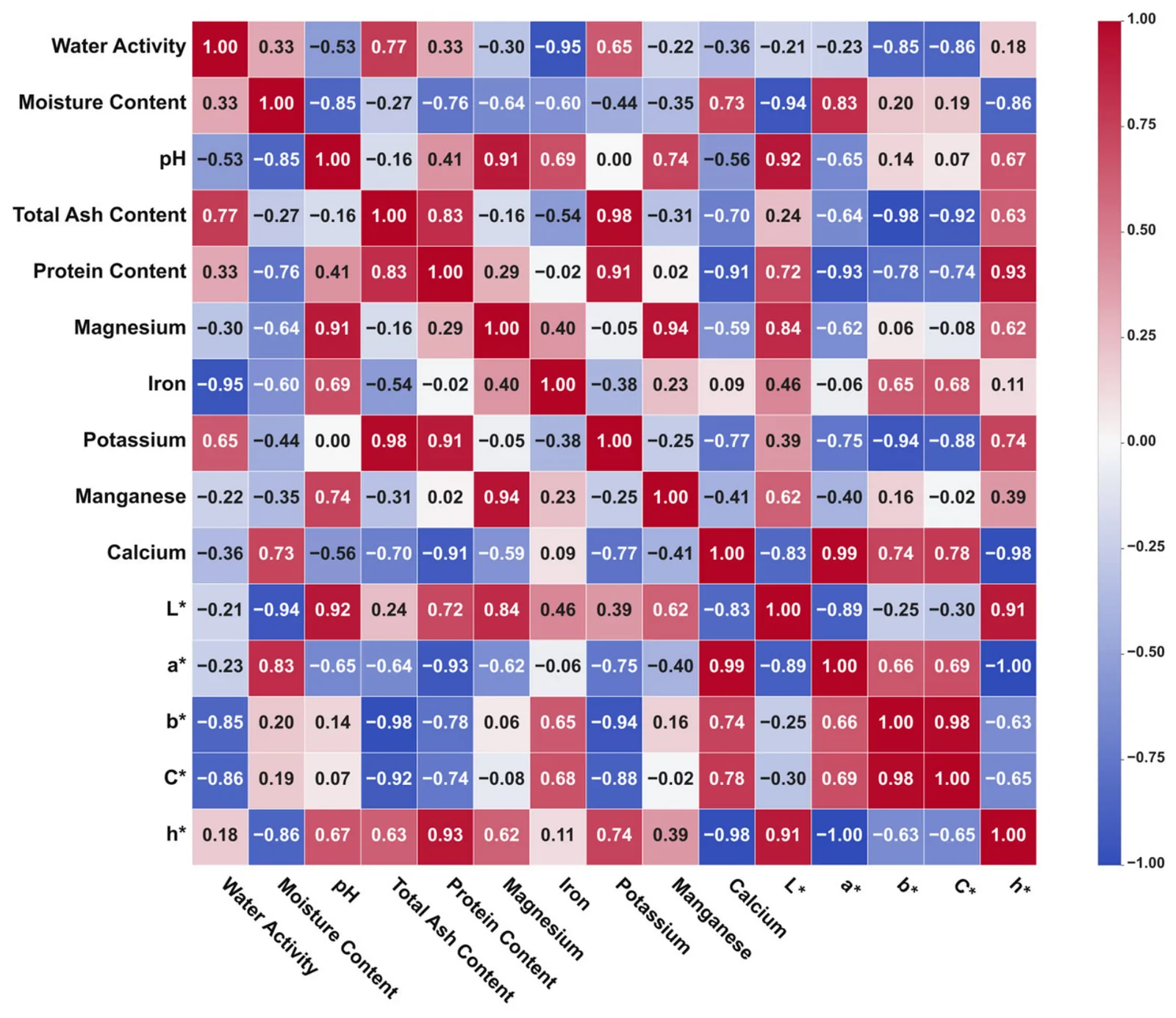
Heatmap of Pearson correlation coefficients of physicochemical parameters. The colour scale ranges from dark blue (strong negative correlation, −1), white (no correlation, values close to zero), to dark red (strong positive correlation, 1). Parameters include water activity, moisture content, pH, total ash content, protein content, magnesium, iron, potassium, manganese, calcium and colour parameters (L*, a*, b*, C* and h*).

Pearson’s correlation was used as an exploratory tool to describe how physicochemical characteristics, mineral composition, and colour attributes co-varied among the mangrove leaf powders. This integrated view may help to identify traits that may serve as complementary markers of species- and maturity-related raw-material variation and supports the future standardisation of botanical materials used for extract preparation (Figure 2). A weak positive relationship was found between water activity (a_w_) and moisture content (r = 0.33). This finding is in accordance with previous research which states that water activity and moisture content do not always correlate linearly, depending on the structural features of the product and moisture sorption properties. According to Barbosa-Cánovas et al. [32], water activity is responsible only for free water available for biochemical and microbiological reactions. In contrast, moisture content showed a strong negative correlation with pH (r = −0.85). This means that the higher the moisture level in samples, the higher their acidity. Possible reasons for this observation may be related to the production of acidic products stimulated by enzymes or microorganisms [33]. Strong positive relationships (r = 0.83) were found between ash and protein contents. The correlation between ash and protein can be explained by increased concentrations of nutrients in these samples since mineral-rich plant products usually contain high concentrations of protein [34]. The second pair are related through their physiological roles in metabolism and osmotic balance [35,36]. In contrast, iron demonstrated weak negative relationships with potassium, probably due to competitive uptake (r = −0.38). Finally, manganese and calcium showed a negative correlation (r = −0.59), which can be explained by different physiological or biochemical roles of these elements [37]. Correlations between the following pairs of colour and physicochemical characteristics were also found lightness (L*) and moisture (r = −0.94)—light samples are characterised by increased reflection [38]; a* and potassium (r = −0.75); b* and iron (r = 0.65) and potassium (r = −0.94)—mineral effects are evident in the development of pigmentation and colour [38].

### 3.3. FT-MIR Monitoring of Oxidative Stability in Linseed Oil Supplemented with Mangrove Extracts

The FT-MIR spectra of linseed oil with synthetic antioxidants and mangrove extracts were studied within the wavenumber interval 4000–600 cm^−1^ to assess the influence of storage on the spectra of these oils by means of the Schaal oven technique [39]. Interpretation focused on intervals showing consistent storage- and treatment-related variation. The 1200–1150 cm^−1^ interval is dominated by C–O and ester-related vibrations of the lipid matrix and was treated as an oxidation-sensitive C–O/ester region [40,41]. Likewise, the broad O–H stretching region can respond to oxidation-related changes and may contain contributions from other hydroxyl-containing compounds, hydrogen-bonded species, and moisture [42,43]. The 1742–1720 cm^−1^ interval covers the carbonyl envelope of the oil matrix, including the ester carbonyl absorption of triacylglycerols; storage-related changes in this interval may be compatible with chemical modification during oxidation [40,41]. Similar O-H and carbonyl changes were observed in linseed oil oxidation processes [42,43]. The wavenumber interval 1532–1510 cm^−1^ was associated with aromatic compound vibrations [44]. Changes in the intensities of these vibrations in the course of oxidation are taken into account as experimental data together with conventional oxidation indices [4].

#### 3.3.1. FT-MIR Characterisation of Oxidation Behaviour in Control Linseed Oil Systems

**Figure 3:**
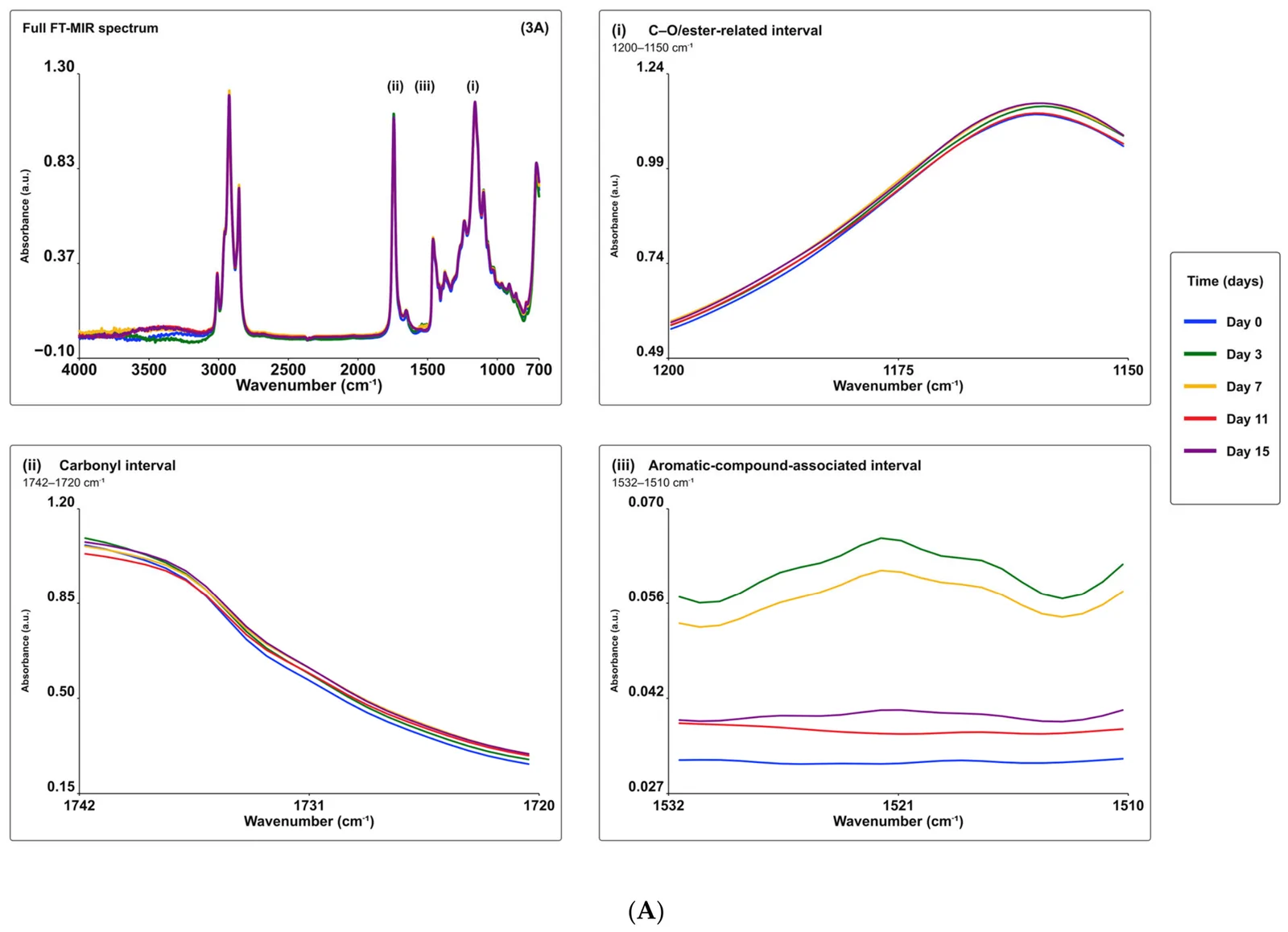

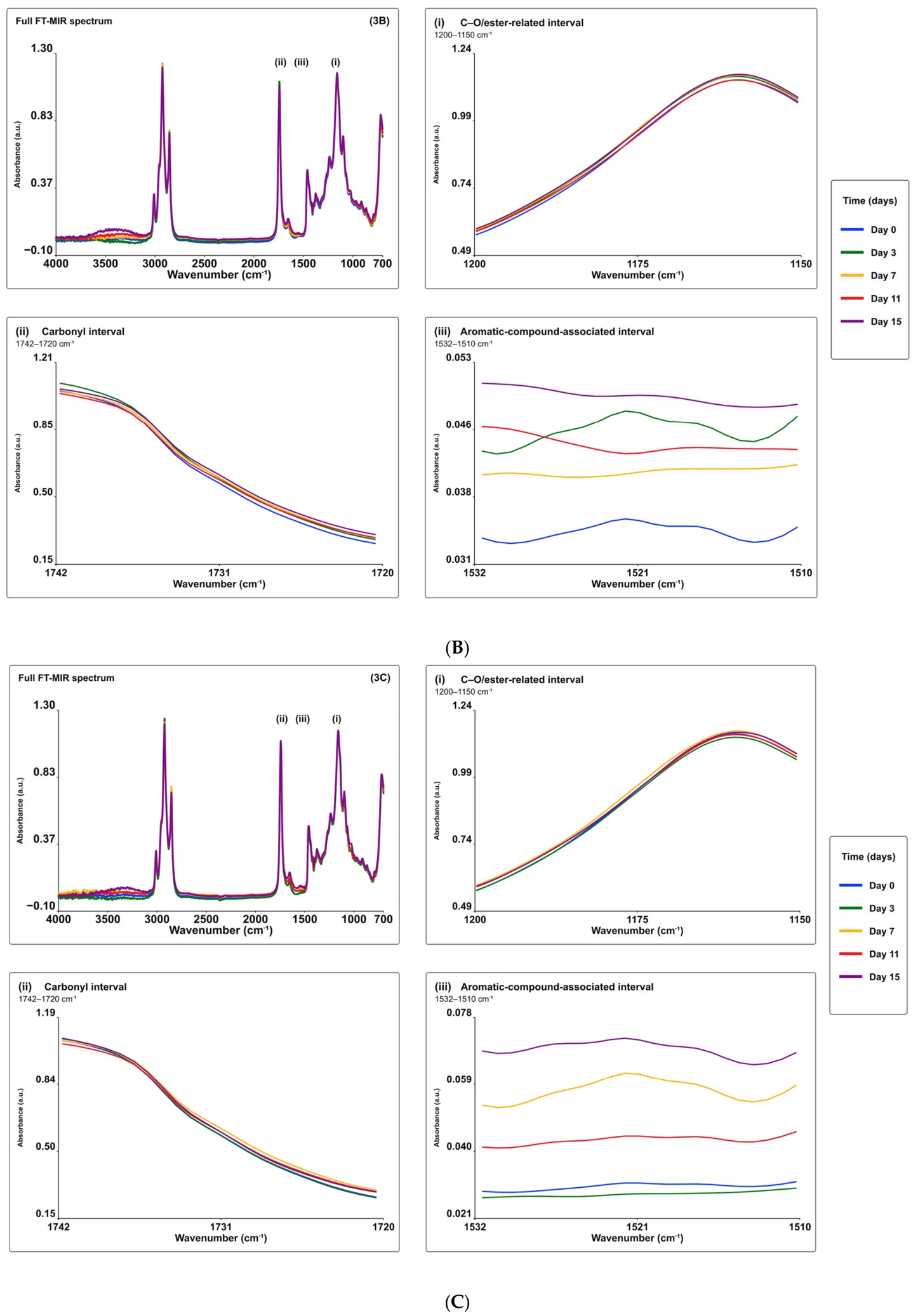

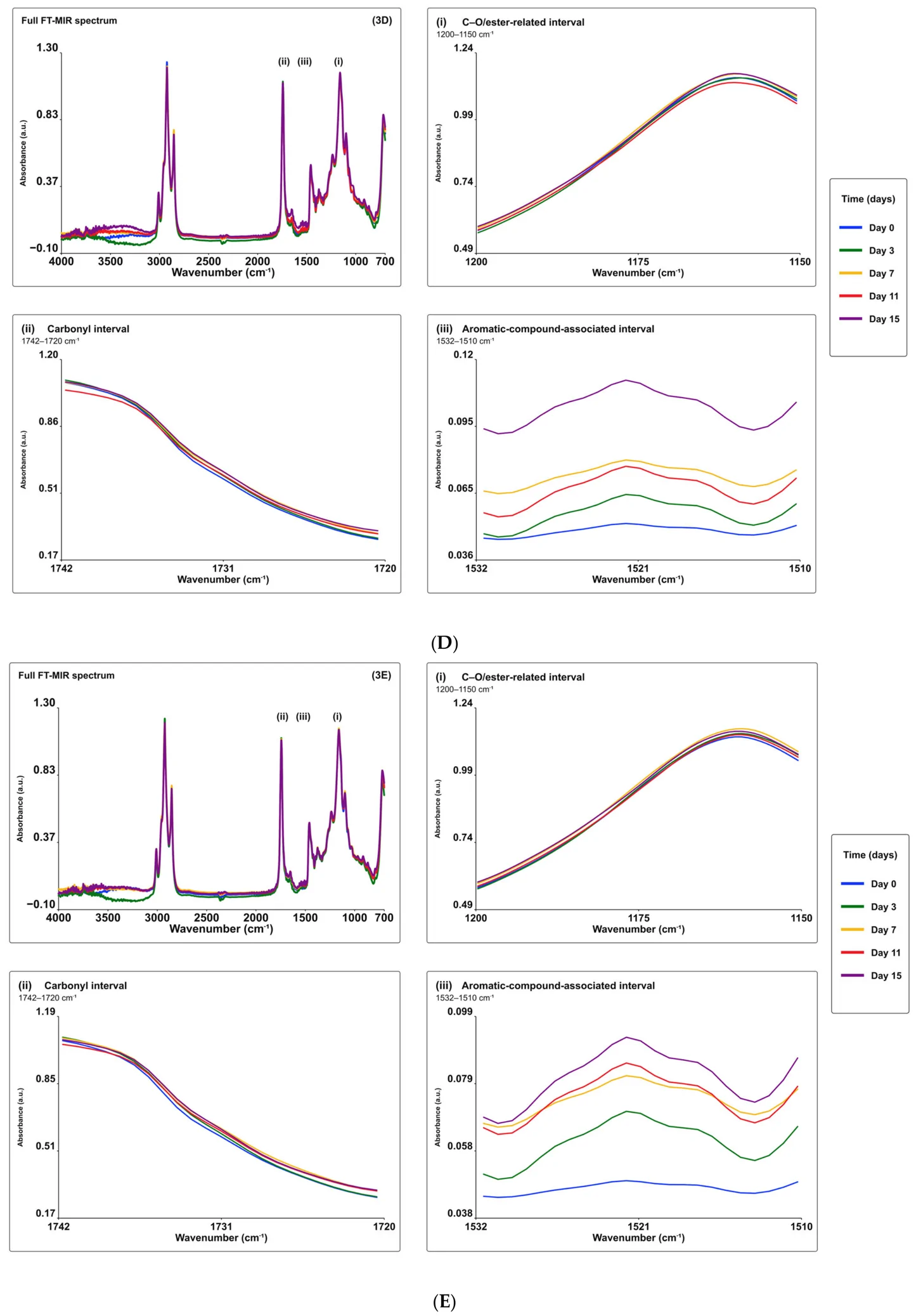

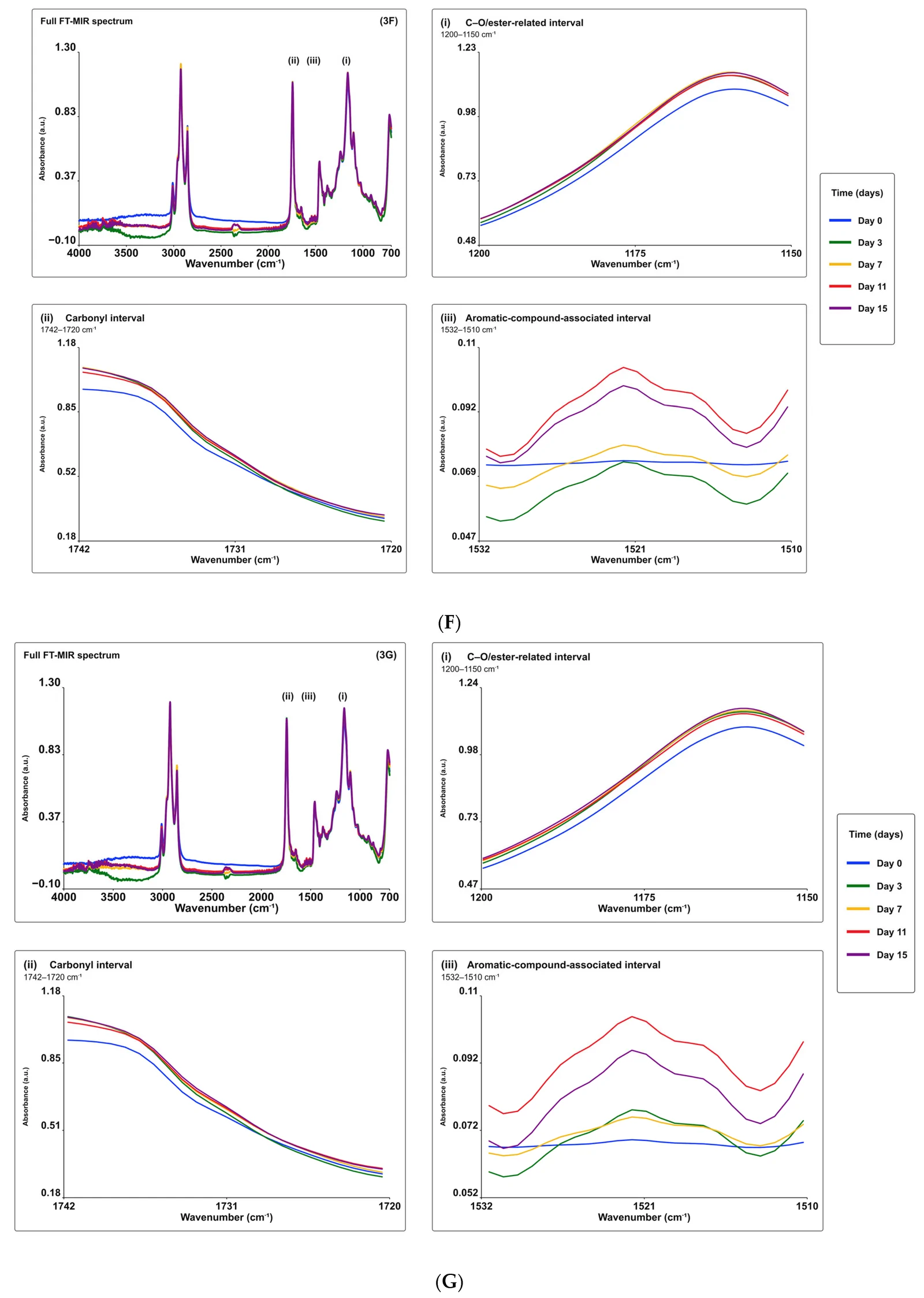
(**A**) FT-MIR spectra of untreated linseed oil (NEG) over storage. Insets (**i**–**iii**) show the expanded spectral regions. (**B**) FT-MIR spectra of BHT-treated linseed oil (POS) over storage. Insets (**i**–**iii**) show the expanded spectral regions. (**C**) FT-MIR spectra of rosemary-treated linseed oil (ROS) over storage. Insets (**i**–**iii**) show the expanded spectral regions. (**D**) FT-MIR spectra of linseed oil treated with young *A. marina* leaf extract (AJE). Insets (**i**–**iii**) show the expanded spectral regions. (**E**) FT-MIR spectra of linseed oil treated with mature *A. marina* leaf extract (AME). Insets (**i**–**iii**) show the expanded spectral regions. (**F**) FT-MIR spectra of linseed oil treated with young *R. mucronata* leaf extract (RJE). Insets (**i**–**iii**) show the expanded spectral regions. (**G**) FT-MIR spectra of linseed oil treated with mature *R. mucronata* leaf extract (RME). Insets (**i**–**iii**) show the expanded spectral regions.

The negative control (NEG), or untreated sample, showed the highest oxidation levels among all the samples and controls on day 11 (Figure 3A). The C–O/ester-related spectral interval (1200–1150 cm^−1^) showed increased absorbance, consistent with oxidation-related changes (Figure 3(Ai)). More evidence was provided by the carbonyl interval (1742–1720 cm^−1^) with increased absorbance, in agreement with the change in the carbonyl envelope (Figure 3(Aii)). This phenomenon occurs due to the decomposition of hydroperoxides into secondary oxidation products. The band associated with the aromatic compound (1532–1510 cm^−1^) showed low absorbance (Figure 3(Aiii)). Although linseed oil contains trace amounts of polyphenols, which may afford some antioxidative protection, it has a strong tendency to oxidise because it contains a high proportion of unsaturated fatty acids, specifically alpha-linolenic acid, which reacts readily with oxygen, heat or light to undergo lipid peroxidation [45]. The effectiveness of polyphenols in linseed oil is greatly restricted by the lack of a robust regeneration mechanism and susceptibility to degradation under oxidative stress [46]. These inherent polyphenols, therefore, are in no way able to counteract the rapid accumulation of primary and secondary oxidation products, leading to rancidity and deterioration in quality. The direct observation is the relative day-11 absorbance change; its oxidation-related interpretation is supported by the matched conventional indices reported for the same oil batches [4].

The positive control (POS), namely BHT, showed moderate antioxidant activity on day 11 (Figure 3B). In the 1200–1150 cm^−1^ C–O/ester-related spectral interval (Figure 3(Bi)), the absorbance was slightly higher than that of rosemary (ROS) but considerably lower than that of the negative control (NEG). This indicated a lower oxidation-sensitive spectral response relative to NEG; however, this interval does not directly quantify hydroperoxide formation [47]. Its FT-MIR profile showed lower oxidation-related absorbance than NEG under the same storage conditions. The carbonyl interval (1742–1720 cm^−1^) (Figure 3(Bii)) absorption was marginally lower than that of the negative control (NEG), suggesting some suppression of oxidation products. Absorbance in the aromatic-compound-associated interval region (1532–1510 cm^−1^) (Figure 3(Biii)) for the positive control (POS) was greater than that for the negative control (NEG). This behaviour can be explained by the protective effect of BHT in stabilising polyphenols in the linseed oil [47]. For the negative control (NEG), where no external antioxidant was added, the polyphenols were degraded more rapidly. This explains the lowest absorbance in the aromatic-compound-associated interval (1532–1510 cm^−1^). BHT acted as a stabilising agent that retarded the degradation of these intrinsic polyphenols when added to the linseed oil. This demonstrates the ability of BHT to scavenge free radicals and indirectly protect the existing antioxidants within the linseed oil, although its inability to regenerate antioxidant capacity remains a limitation [48]. These observations describe differences in spectral trajectories and do not directly measure BHT concentration, residual antioxidant activity or individual oxidation products.

The rosemary-supplemented linseed oil (ROS) showed lower day-11 absorbance than NEG in the 1200–1150 cm^−1^ interval (Figure 3(Ci)). The carbonyl interval (1742–1720 cm^−1^) (Figure 3(Cii) further showed rosemary’s ability to inhibit oxidation products. The aromatic-compound-associated interval (1532–1510 cm^−1^) suggested residual presence of active phenolic compounds (Figure 3(Ciii)), which retard lipid peroxidation through redox cycling and metal-chelating properties. These results underline the synergistic action of rosemary phenolics, which were more effective than BHT in stabilising oxidative processes [49]. The spectrum alone cannot establish the identity or persistence of rosemary phenolics; this would require targeted compositional analysis.

Across the control systems, these between-treatment absorbance differences are reported as relative oxidation-sensitive spectral responses, not as direct evidence of a specific antioxidant effect or compound concentration.

#### 3.3.2. FT-MIR Characterisation of Oxidation Behaviour in Mangrove Extract-Treated Linseed Oil

Linseed oil supplemented with young *A. marina* leaf extracts (AJE) exhibited moderate antioxidant activity (Figure 3D), as indicated by the absorbance observed in the C–O/ester-related spectral interval (1200–1150 cm^−1^) (Figure 3(Di)) on day 11, which was lower than that of the negative control (NEG) but higher than those of the positive control (POS) and rosemary (ROS). This result suggests a partial suppression of primary oxidation product formation. Similarly, the absorbance in the aldehyde region (1742–1720 cm^−1^) indicated limited efficacy in the mitigation of secondary oxidation products (Figure 3(Dii)). The absorbance observed in the carbonyl interval (1532–1510 cm^−1^) was treated as an aromatic-compound-associated feature (Figure 3(Diii)). However, the oxidative degradation of these polyphenols during storage and the presence of some trace metals, e.g., Fe^2+^ and Cu^2+^, may instead promote pro-oxidant reactions through Fenton-like mechanisms [50]. The Fenton reaction involves the catalytic conversion of hydrogen peroxide into hydroxyl radicals (OH) by transition metals like Fe^2+^, which can initiate or propagate lipid oxidation, especially in the presence of unsaturated fatty acids [51]. This spectral response cannot be used to infer the concentration or antioxidant activity of individual phenolic classes.

On the other hand, mature leaf extracts of *A. marina* (AME) demonstrated the highest antioxidant activity (Figure 3E) in line with low absorption in the C–O/ester-related interval (1200–1150 cm^−1^) (Figure 3(Ei)) and carbonyl interval (1742–1720 cm^−1^) on day 11 compared with the other samples and controls (Figure 3(Eii)). Although, spectral difference does not quantify individual phenolic compounds, the absorbance in the polyphenol region (1532–1510 cm^−1^) was higher than that of young leaf extracts of *A. marina* (AJE) (Figure 3(Eiii)). These compounds have been reported to possess strong radical-scavenging and metal-chelating abilities, which can efficiently neutralise reactive oxygen species (ROS) and inhibit trace metal-catalysed oxidation. The chelation of pro-oxidant metals by these compounds interrupts the catalytic cycle of lipid oxidation, reducing the formation of highly reactive radicals and stabilising the oil matrix [42,50]. Despite these advantages, the degradation of phenolics into less active intermediates over time may limit long-term stabilisation. The association of spectral features with AME should therefore be interpreted alongside the conventional oxidation results for the same oil batches [4], rather than as evidence of a specific phenolic mechanism.

Linseed oil containing young leaf extracts of *R. mucronata* (RJE) displayed moderate antioxidant behaviour (Figure 3F), with higher absorbance in the C–O/ester-related interval (1150–1200 cm^−1^ (Figure 3(Fi)) and similar values in the carbonyl interval (1742–1720 cm^−1^), in comparison to the negative control (NEG) on day 11 (Figure 3(Fii)). The absorbance in the aromatic-compound-associated band (1532–1510 cm^−1^) was the highest among the samples and controls (Figure 3(Fiii)), reflecting a substantial phenolic content. However, catechins and phenolic acids in RJE may degrade into pro-oxidant intermediates under prolonged oxidative stress, contributing to the higher oxidation product levels [50]. Such degradation may occur due to the oxidation of phenolics into quinone-like structures, which can react with oxygen or lipid radicals, exacerbating oxidative stress [51]. This behaviour underscores the potential of polyphenols to exhibit dual antioxidant and pro-oxidant roles depending on environmental conditions. Environmental factors such as temperature, the presence of trace metals, and oxygen concentration can significantly influence these dual roles, with trace metals like Fe^2+^ or Cu^2+^ catalysing Fenton-like reactions that promote lipid peroxidation [50]. Because individual phenolics were not quantified, this pattern does not establish the behaviour of catechins or particular phenolic acids.

In contrast, mature leaf extract of *R. mucronata* (RME) in linseed oil showed a unique profile (Figure 3G), with aromatic-compound-associated band (1532–1510 cm^−1^) absorbance (Figure 3(Giii)) close to that of RJE but higher in carbonyl interval (1742–1720 cm^−1^) absorption (Figure 3G(ii)), indicating incomplete suppression of oxidation products. The C–O/ester-related interval (1200–1150 cm^−1^) absorption was lower than that of RJE but higher than that of AME (Figure 3(Giii)). This suggests that while RME’s higher phenolic content provides robust antioxidant activity, interactions with trace metals or oxidative degradation may reduce its long-term efficacy [51]. The phenolics in RME, such as ellagic acid and flavonoids, are potent antioxidants capable of scavenging radicals and chelating trace metals. However, the incomplete neutralisation of oxidation products may arise from the competitive binding of these phenolics with both radicals and metals, potentially reducing their overall efficacy [50]. The relative band intensities demonstrate spectral differences, whereas the proposed roles of metals or individual phenolics remain hypotheses for targeted chemical analysis.

#### 3.3.3. Effect of Leaf Maturity on Phenolic Composition and Antioxidant Performance

Leaf maturity is an important factor influencing the level of antioxidant activity in mangrove leaf extracts. Mature *A. marina* and *R. mucronata* leaves showed higher levels of protective activity against oxidative stress. Mature leaves contain higher flavonoid and tannin contents, which have been found to have remarkable radical-scavenging and metal-chelating activities [50]. These compounds have been observed to repress the formation of reactive oxygen species (ROS) and break lipid peroxidation chain reactions, making immobilised lipid matrices. The FT-MIR findings corroborate the oxidation trends previously reported by Singh et al. [4] for the same linseed-oil batches. The higher concentrations of phenolics in mature leaf samples were attributed to their prolonged susceptibility to various environmental stresses, which triggered the induction of stress metabolism [51]. Younger leaf samples, however, focusing on their primary metabolism, resulted in lower antioxidant activities. Nevertheless, high phenolic loads can display pro-oxidant behaviour under severe oxidative conditions, as reflected by elevated aldehydes in mature *R. mucronata* leaves despite intense aromatic-compound-associated band (1510–1532 cm^−1^), highlighting the conditional duality of polyphenols [50]. The observed association with leaf maturity does not demonstrate a causal effect of maturity or phenolic composition on the FT-MIR response.

Likewise, comparisons among extract-treated oils describe differences in storage-related spectra and do not, without compositional measurements, establish a mechanism involving individual phenolics or metals.

### 3.4. Chemometric Tools Applied to FT-MIR Spectra

**Figure 4:**
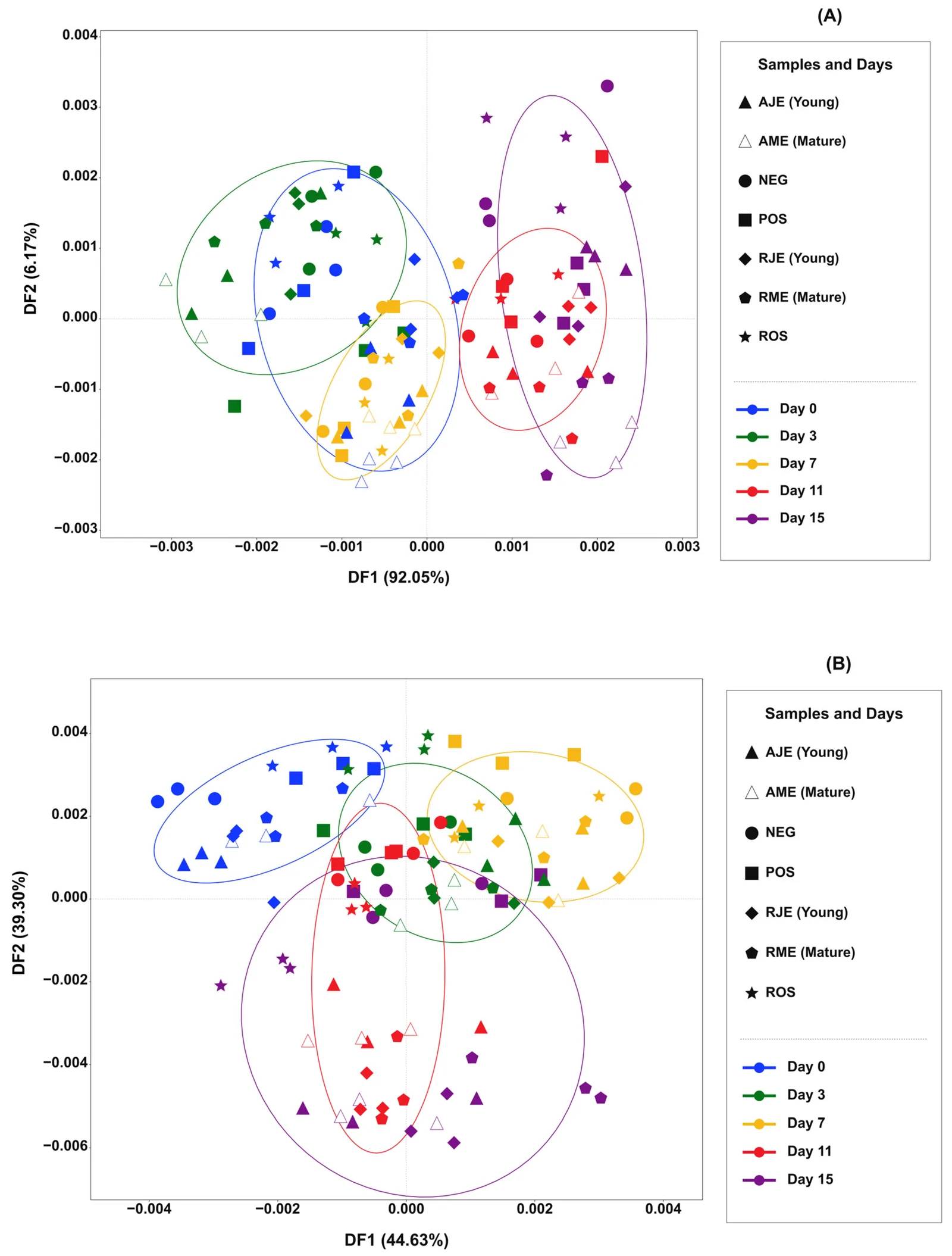

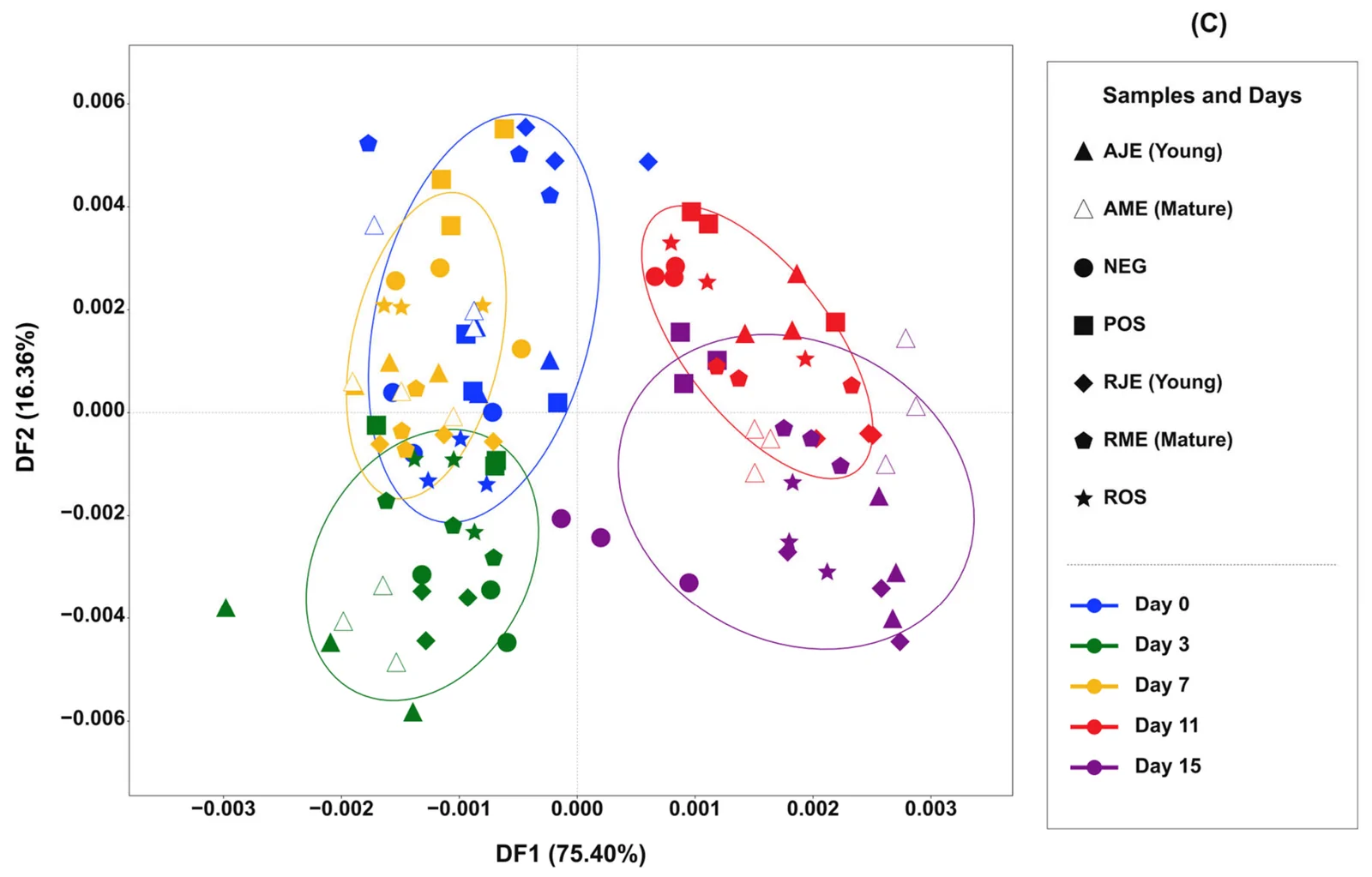
(**A**) Factorial discriminant analysis similarity map for the 1200–1150 cm^−1^ region. Storage days and treatment symbols are identified in the legend. (**B**) Factorial discriminant analysis similarity map for the 1742–1720 cm^−1^ region. Storage days and treatment symbols are identified in the legend. (**C**) Factorial discriminant analysis similarity map for the concatenated spectral regions. Storage days and treatment symbols are identified in the legend.

The FDA score plot applied to the C–O/ester-related spectral interval is shown in Figure 4A. The observations from the five storage days formed partially distinct, but overlapping, groups. The partial overlap indicates limited separation of storage days using this oxidation-sensitive spectral region. Consequently, the hydroperoxide band alone did not fully separate all stages of ageing. The leave-one-spectrum-out cross-validation accuracy was 58.10% (Table 2A), with the greatest overlap occurring among the early storage classes. The FDA model was used to describe internal separation of storage-related spectra; it does not quantify hydroperoxides or rank antioxidant efficacy independently of the conventional data.

Despite this moderate classification performance, Figure 4A provides useful information. The NEG samples followed the expected progression away from the initial spectral state as storage proceeded, in agreement with the strong C–O/ester-related absorbance reported for untreated oil in Section 3.3.1. The treated oils showed treatment-dependent distributions within the day clusters. This indicates treatment-dependent spectral variation within the present dataset. The result is consistent with the FT-MIR observations indicating that BHT and AJE provided partial protection, whereas rosemary and AME more strongly limited the development of primary oxidation products [4].

Figure 4B presents the FDA score plot for the carbonyl interval (1742–1720 cm^−1^), which covers the carbonyl envelope of the oil matrix. This model gave a higher leave-one-spectrum-out accuracy of 71.43% (Table 2B), indicating that the carbonyl region contained more discriminating information than the C–O/ester-related interval. In particular, day 3 and day 7 were classified with 95.24% and 100% accuracy, respectively. The lower correct classification of day 0, day 11 and day 15 reflects overlap between the advanced storage stages, where hydroperoxide decomposition and subsequent secondary reactions may produce related spectral profiles.

This behaviour agrees with the interpretation in Section 3.3 and Section 3.3.1. The untreated NEG oil showed the strongest oxidation response around day 11, whereas the antioxidant-treated oils displayed modified spectral trajectories. The separation of treated samples from the NEG pattern indicates treatment-dependent spectral trajectories. It does not, by itself, establish which treatment was best; rather, it shows that the treatments produced measurably different oxidation-related spectral responses. This interpretation is consistent with the lower PV, TBARS, acid index, and p-anisidine values previously reported for antioxidant-treated oils relative to NEG [4].

Finally, the combined model in Figure 4C was constructed by concatenating the first 5 principal components of the PCA applied separately to C–O/ester-related interval and carbonyl interval. This approach gave the highest leave-one-spectrum-out classification accuracy, 76.19% (Table 2C). DF1 accounted for 75.40% of the variance shown in the score plot, while DF2 accounted for 16.36% of the variance. The clearer separation of the early storage observations from the day 11 and day 15 observations shows that combining information from oxidation stages improves the description of the ageing process. The practical value of this model is that it links the two spectral events that occur during lipid oxidation. Hydroperoxides accumulate during the early stage and later give rise to aldehydes and other secondary products. Considering both regions together, therefore captures more the oxidation pathway than either region alone. The day-wise clusters in Figure 4C show a consistent progression from the early storage period towards the advanced ageing period, while the treatment symbols indicate that the oils did not all follow the same trajectory.

When interpreted together with Section 3.3, the concatenated FDA model provides additional evidence of treatment-dependent spectral trajectories in linseed oil. The NEG samples represent the untreated oxidation trajectory, while BHT, rosemary, and the mangrove extracts show altered positions within the storage-day clusters. These differences are compatible with the lower oxidation-product absorbance observed for rosemary and particularly AME and with the more variable behaviour of the *R. mucronata* treatments. The FDA results should therefore be presented as chemometric support for the FT-MIR and conventional oxidation findings, rather than as an independent proof of antioxidant ranking. The improved separation after concatenation approach reflects complementary information in the two selected spectral intervals but does not quantify primary or secondary oxidation products directly. These findings are in agreement with previous findings reporting that analysing different data tables simultaneously allowed better characterisation of food products [52].

## 4. Conclusions

This study evaluates mangrove leaf extracts in a linseed-oil model using physicochemical characterisation and targeted FT-MIR monitoring. Physicochemical characterisation of *A. marina* and *R. mucronata* leaves showed distinct compositional differences, with young *A. marina* containing the highest protein level. All samples exhibited high potassium and magnesium contents, particularly *A. marina*, describing compositional variation among the leaf powders.

FT-MIR analysis showed treatment-dependent oxidation-related spectral changes. During the Schaal oven test, treated oils showed distinct spectral trajectories relative to the controls. Chemometric evaluation using FDA showed that the carbonyl interval provided stronger discrimination of storage-related spectral changes than the C–O/ester-related interval (71.43% versus 58.10%), while concatenation of both oxidation-related regions improved the overall accuracy to 76.19%. These results provide complementary chemometric support for the FT-MIR evidence of treatment-dependent preservation and are consistent with the conventional oxidation data reported by Singh et al. [4], particularly the superior stabilising effect of mature *A. marina* extract. The FDA findings are therefore interpreted as complementary evidence of storage-related spectral discrimination, rather than direct confirmation of a specific oxidation product or antioxidant mechanism.

Overall, FT-MIR spectroscopy coupled with chemometric tools provide a rapid approach for monitoring oxidation-related spectral changes in the present linseed-oil model. Mangrove leaf-derived extracts merit further investigation as potential natural preservation materials; however, their elemental safety, including Pb, Cd, Hg and As, toxicological profile, bioactive composition and sensory performance should be established in relevant food matrices before food-use suitability or real-world application can be evaluated.

## Figures and Tables

**Table 1:** (**A**) Physicochemical parameters of young and mature mangrove leaf samples. (**B**) Mineral composition of young and mature mangrove leaf samples. (**C**) Colour parameters of young and mature mangrove leaf samples.

**(** **A)**					
**Species**	**Water Activity (a_w_)**	**Moisture Content (g/100 g)**	**pH**	**Ash Content (g/100 g)**	**Protein Content (g/100 g)**
*A. marina* (Young leaf)	0.386 ± 0.00 ^a^	1.05 ± 0.02 ^b^	5.357 ± 0.01 ^c^	13.15 ± 0.05 ^a^	11.07 ± 0.02 ^a^
*R. mucronata* (Young leaf)	0.384 ± 0.00 ^a^	1.24 ± 0.02 ^a^	5.007 ± 0.00 ^d^	10.400 ± 0.14 ^b^	4.14 ± 0.10 ^d^
*A. marina* (Mature leaf)	0.373 ± 0.0 ^b^	1.023 ± 0.04 ^b^	5.937 ± 0.00 ^a^	10.466 ± 0.03 ^b^	8.327 ± 0.1 ^b^
*R. mucronata* (Mature leaf)	0.362 ± 0.00 ^c^	1.085 ± 0.04 ^ab^	5.510 ± 0.02 ^b^	9.384 ± 0.06 ^c^	5.533 ± 0.008 ^c^
(**B**)					
**Species**	**Mg (mg/L)**	**Fe (mg/L)**	**K (mg/L)**	**Mn (mg/L)**	**Ca (mg/L)**
*A. marina* (Young leaf)	35.33 ± 0.22 ^b^	1.385 ± 0.02 ^c^	101.01 ± 1.52 ^a^	tr	39.11 ± 1.54 ^a^
*A. marina* (Mature leaf)	45.115 ± 0.13 ^a^	1.655 ± 0.00 ^b^	76.04 ± 0.21 ^b^	1.115 ± 0.00 ^a^	39.48 ± 0.33 ^a^
*R. mucronata* (Young leaf)	34.015 ± 0.11 ^c^	1.22 ± 0.01 ^d^	69.04 ± 0.58 ^c^	0.53 ± 0.01 ^b^	ND
*R. mucronata* (Mature leaf)	35.91 ± 0.06 ^b^	1.885 ± 0.02 ^a^	64.795 ± 0.19 ^c^	tr	ND
(**C**)					
**Species**	L*	a*	b*	C*	h*
*A. marina* (Young leaf)	54.08 ± 0.008 ^b^	−0.35 ± 0.003 ^c^	23.24 ± 0.005 ^d^	23.73 ± 0.003 ^d^	90.85 ± 0.005 ^b^
*R. mucronata* (Young leaf)	52.20 ± 0.003 ^d^	7.04 ± 0.003 ^a^	26.76 ± 0.005 ^b^	26.67 ± 0.006 ^b^	75.24 ± 0.006 ^d^
*A. marina* (Mature leaf)	55.11 ± 0.003 ^a^	−0.42 ± 0.003 ^d^	26.59 ± 0.008 ^c^	26.11 ± 0.008 ^c^	90.92 ± 0.008 ^a^
*R. mucronata* (Mature leaf)	53.43 ± 0.003 ^c^	5.79 ± 0.003 ^b^	29.41 ± 0.003 ^a^	29.98 ± 0.003 ^a^	78.85 ± 0.005 ^c^

All data are presented as means ± standard deviation (*n* = 3). Mean values with different superscripts in the same column are statistically significantly different (*p* < 0.05) according to Fisher’s test. Mg, magnesium; Mn, manganese; Cu, copper; Zn, zinc; K, potassium; Ca, calcium; Fe, iron; tr, traces; Zn and Cu were found at trace concentrations, i.e., <0.50 mg/L; ND, not detectable. L* = Lightness, a* = Redness (+a*) to Greenness (−a*), b* = Yellowness (+b*) to Blueness (−b*), C* = chroma and h* = Hue angle.

**Table 2:** (**A**) Leave-one-out cross-validation classification for the 1200–1150 cm^−1^ region. (**B**) Leave-one-out cross-validation classification for the 1742–1720 cm^−1^ region. (**C**) Leave-one-out cross-validation classification for the concatenated spectral regions.

**(** **A)**							
**Observed/Predicted**	**Day 0**	**Day 3**	**Day 7**	**Day 11**	**Day 15**	**Total**	**% Correct**
Day 0	1	9	8	3	0	21	4.76%
Day 3	6	14	1	0	0	21	66.67%
Day 7	4	0	16	1	0	21	76.19%
Day 11	0	0	0	15	6	21	71.43%
Day 15	0	0	0	6	15	21	71.43%
Total	11	23	25	25	21	105	58.10%
(**B**)							
**Observed/Predicted**	**Day 0**	**Day 3**	**Day 7**	**Day 11**	**Day 15**	**Total**	**% Correct**
Day 0	15	5	0	1	0	21	71.43%
Day 3	1	20	0	0	0	21	95.24%
Day 7	0	0	21	0	0	21	100%
Day 11	2	0	2	7	10	21	33.33%
Day 15	2	0	3	4	12	21	57.14%
Total	20	25	26	12	22	105	71.43%
(**C**)							
**Observed/Predicted**	**Day 0**	**Day 3**	**Day 7**	**Day 11**	**Day 15**	**Total**	**% Correct**
Day 0	14	3	3	1	0	21	66.67%
Day 3	2	19	0	0	0	21	90.48%
Day 7	3	0	18	0	0	21	85.71%
Day 11	0	0	0	14	7	21	66.67%
Day 15	0	2	0	4	15	21	71.43%
Total	19	24	21	19	22	105	76.19%

## Data Availability

The original contributions presented in this study are included in the article. Further inquiries can be directed to the corresponding author.
